# Characterization of *TaSPP-5A* gene associated with sucrose content in wheat (*Triticum aestivum* L.)

**DOI:** 10.1186/s12870-022-03442-x

**Published:** 2022-02-01

**Authors:** Fanli Jing, Yongping Miao, Peipei Zhang, Tao Chen, Yuan Liu, Jingfu Ma, Mengfei Li, Delong Yang

**Affiliations:** 1 Gansu Provincial Key Lab of Aridland Crop Science, Lanzhou, 730070 Gansu China; 2 College of Life Science and Technology, Gansu Agricultural University, Lanzhou, 730070 Gansu China

**Keywords:** Wheat, Thousand-grain weight, Sucrose content, *TaSPP*, Allelic variation, Functional marker

## Abstract

**Background:**

Sucrose, the major product of photosynthesis and the primary sugar transported as a soluble carbohydrate via the phloem, is a critical determinant for harvest yield in wheat crops. Sucrose-phosphatase (SPP) catalyzes the final step in the sucrose biosynthesis pathway, implying its essential role in the plant.

**Result:**

In this study, wheat *SPP* homologs genes were isolated from chromosomes 5A, 5B, and 5D, designated as *TaSPP-5A*, *TaSPP-5B*, and *TaSPP-5D*, respectively. Sequence alignment showed one 1-bp Insertion-deletion (InDel) and three single nucleotide polymorphisms (SNPs) at *TaSPP-5A* coding region, forming two haplotypes, *TaSPP-5Aa* and *TaSPP-5Ab*, respectively. A derived cleaved amplified polymorphism sequence (dCAPS) marker, *TaSPP-5A*-dCAPS, was developed to discriminate allelic variation based on the polymorphism at position 1242 (C-T). A total of 158 varieties were used to perform a *TaSPP-5A* marker-trait association analysis, where two haplotypes were significantly associated with sucrose content in two environments and with thousand-grain weight (TGW) and grain length (GL) in three environments. Quantitative real-time PCR further revealed that *TaSPP-5Aa* showed relatively higher expression than *TaSPP-5Ab* in wheat seedling leaves, generally associating with increased sucrose content and TGW. The expression of *TaSPP-5A* and sucrose content in *TaSPP-5Aa* haplotypes were also higher than those in *TaSPP-5Ab* haplotypes under both 20% PEG-6000 and 100 μM ABA treatment. Sequence alignment showed that the two *TaSPP-5A* haplotypes comprised 11 SNPs from -395 to -1962 bp at *TaSPP-5A* promoter locus, participating in the formation of several conserved sequences, may account for the high expression of *TaSPP-5A* in *TaSPP-5Aa* haplotypes. In addition, the distribution analysis of *TaSPP-5A* haplotypes revealed that *TaSPP-5Aa* was preferred in the natural wheat population, being strongly positively selected in breeding programs.

**Conclusion:**

According to the SNPs detected in the *TaSPP-5A* sequence, two haplotypes, *TaSPP-5Aa* and *TaSPP-5Ab*, were identified among wheat accessions, which potential value for sucrose content selection was validated by association analysis. Our results indicate that the favorable allelic variation *TaSPP-5Aa* should be valuable in enhancing grain yield by improving the sucrose content. Furthermore, a functional marker, *TaSPP*-*5A*-dCAPS, can be used for marker-assisted selection to improve grain weight in wheat and provides insights into the biological function of *TaSPP-5A* gene.

**Supplementary Information:**

The online version contains supplementary material available at 10.1186/s12870-022-03442-x.

## Background

Wheat (*Triticum aestivum* L.) is one of the most important cereal crops, and breeding new wheat varieties with higher grain production and stronger abiotic stress resistance is required to meet the projected requirements of wheat for an increasing world population. Due to the abundance and relative stability of single nucleotide polymorphisms (SNPs) in the genome, SNPs have been used as molecular markers to identify genes related with observed traits [1–3]. Many genes contributing to grain yield have been identified via SNPs genotyping in wheat, including sucrose synthase (*TaSus2-2B*), *TaGW2*, *TaGW2-6B,* cell wall invertase gene (*TaCwi-A1*), cytokinin oxidase/dehydrogenase (*TaCKX6-D1*), glutamine synthetase (*TaGS1a*), *TaSAP1-A1, TaGASR7-A1, TaGS-D1,* IAA-glucose hydrolase (*TaTGW6*)*,* trehalose 6-phosphate phosphatase (*TaTPP-6AL1*) and sucrose-fructan 6-fructosyltransferase (*6-SFT-A2*) [1, 4–16].

In crop plants, sucrose serves a pivotal role in the distribution of carbon resources, and thus its accumulation and remobilization are critical determinants for harvest yield and grain quality [17]. Sucrose is synthesized in the cytosol by consecutive action of sucrose-phosphate synthase (SPS; EC 2.4.1.14) and sucrose phosphatase (SPP; EC 3.1.3.24) [18, 19]. SPS transfers the glucosyl moiety of UDP glucose onto fructose-6-phosphate to produce sucrose-6-phosphate, which is dephosphorylated by SPP to release free sucrose thereafter. The reaction catalyzed by SPP is essentially irreversible and displaces the reversible SPS reaction from equilibrium into the direction of net sucrose synthesis [18]. SPS has been generally recognized as a key regulator of the whole pathway of sucrose synthesis [20, 21], while the function of SPP in the regulation of assimilate partitioning has been relatively neglected [22]. Early reports showed that SPP activity appeared in large excess over SPS activity, indicating that SPP is unlikely to play a regulatory role in sucrose synthesis [23]. However, several pieces of evidence have revealed that SPP and SPS existed in a complex form in plants with remarkably similar maximum potential activities to each other [24–26]. SPP physically interacts with SPS, providing a new level of regulation of sucrose synthesis [27]. It suggests that SPP could have an essential role in metabolite channeling between the two enzymes and could more contribute to the control of sucrose biosynthesis than previously proposed [22, 26].

SPP belongs to a family of phosphatases/hydrolases with phosphohydrolase and C-terminal domains common in higher plants [28, 29] and is encoded by small gene families [30]. As so far, *SPP* genes have been cloned in different plant species, such as *Arabidopsis thaliana*, tomato (*Lycopersicon esculentum*), rice (*Oryza sativa*), maize (*Zea mays*), barley (*Hordeum vulgare*), and wheat [31–33], where they constitute gene families with different number of members depending on the species. Four genomic *SPP*-like genes are available from *Arabidopsis thaliana* and rice, respectively, which have very similar exon-intron structures [31]. In wheat, three *SPP*-like cDNA (*TaSPP1*, *TaSPP2*, and *TaSPP3*) have been cloned with 94% identity at the nucleotide level with each other, indicative of homologous genes in wheat genomes [31]. The deduced amino acid sequences also show very high similarity with the barley *SPP1*, maize *SPP1*, and rice *SPP1* and *SPP2* sequences [31], suggesting that these are orthologous genes. However, due to the enormous genome size and allohexaploid nature of bread wheat, it is essential to ensure that primer sets for cloning genes are chromosome-specific [34]. Hitherto, the main objectives of this study were to characterize the genomic sequence of *TaSPP*, investigate the haplotype variation of *TaSPP* genes, and perform association analysis using multiple wheat varieties. Additionally, the geographic distribution and frequency of favorable allelic variation of *TaSPP* were analyzed. The purpose is to provide a new and effective molecular marker for breeding high yield wheat variety by marker-assisted selection.

## Results

### Cloning and nucleotide diversity in the region of *TaSPP-5A*

**Fig. 1:**
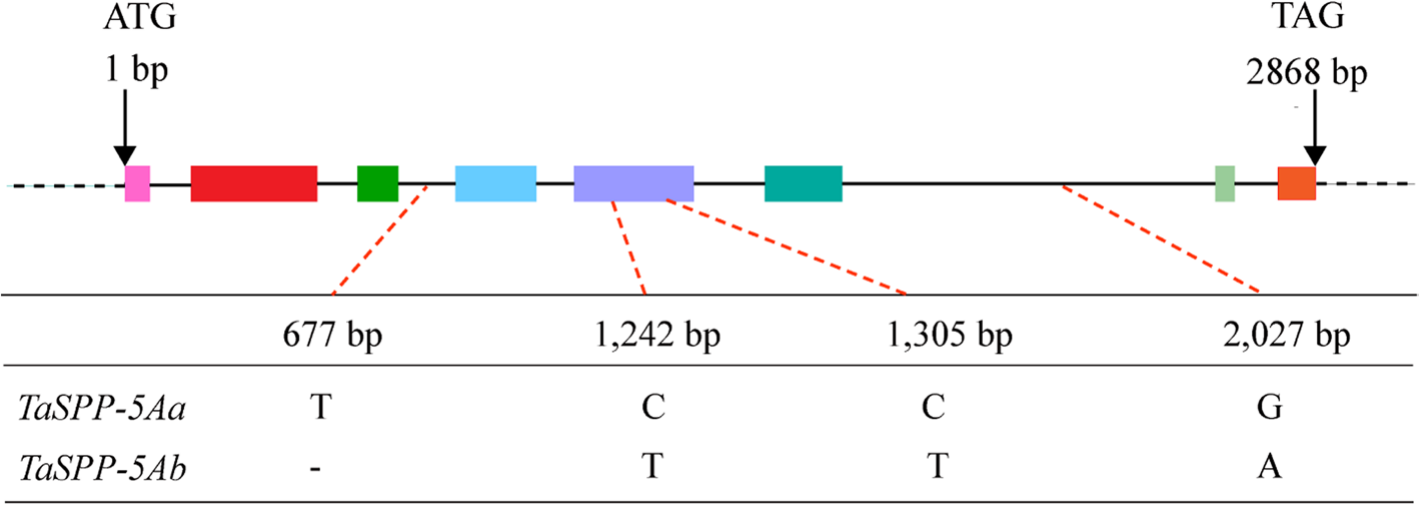
Schematic diagram of *TaSPP-5A* structure, SNP and InDel in two haplotypes identified in *TaSPP-5A* among the wheat diversity panel. Exons were represented by different colored boxes, introns were denoted by blank lines, and UTRs were represented by a black dotted line

To obtain possible reference sequence of *TaSPP* genes, rice *OsSPP* (Os01g0376700) was used as the query against the *Ensembl*Plants (http://plants.ensemble.org/index.html) database. Three *SPP* homologs with high similarity were obtained, named *TaSPP1*, *TaSPP2*, and *TaSPP3* in a previous report [31]. The sequences of three homologous genes were used to perform sequence alignment with the genome sequences of three published wheat varieties, Chinese Spring, Fielder, and Zang 1817, which were accessed at the WheatOmics 1.0 website (http://202.194.139.32/blast/viroblast.php). Sequence alignment showed that the three homologous genes were distributed on 5A, 5B, and 5D chromosomes, respectively. Therefore, they were renamed as *TaSPP-5A*, *TaSPP-5*B, and *TaSPP-5D* herein. The specific primer pairs of *TaSPP-5A*, *TaSPP-5B,* and *TaSPP-5D* genes were used to isolate the target genes in different wheat varieties (Additional file 1: Figure S1; Table S1). A 1-bp Insertion-deletion (InDel) and three single nucleotide polymorphisms (SNPs) at the coding region were identified in *TaSPP-5A*. The InDel and SNPs were located at 677, 1242, 1305, and 2077 bp, forming two haplotypes, *TaSPP-5Aa* and *TaSPP-5Ab*, respectively (Fig. 1). No polymorphisms were detected in *TaSPP-5B* and *TaSPP-5D* in the analysed sequence among the varieties included in this study.

### Development of dCAPS markers in the *TaSPP-5A* gene

**Fig. 2:**
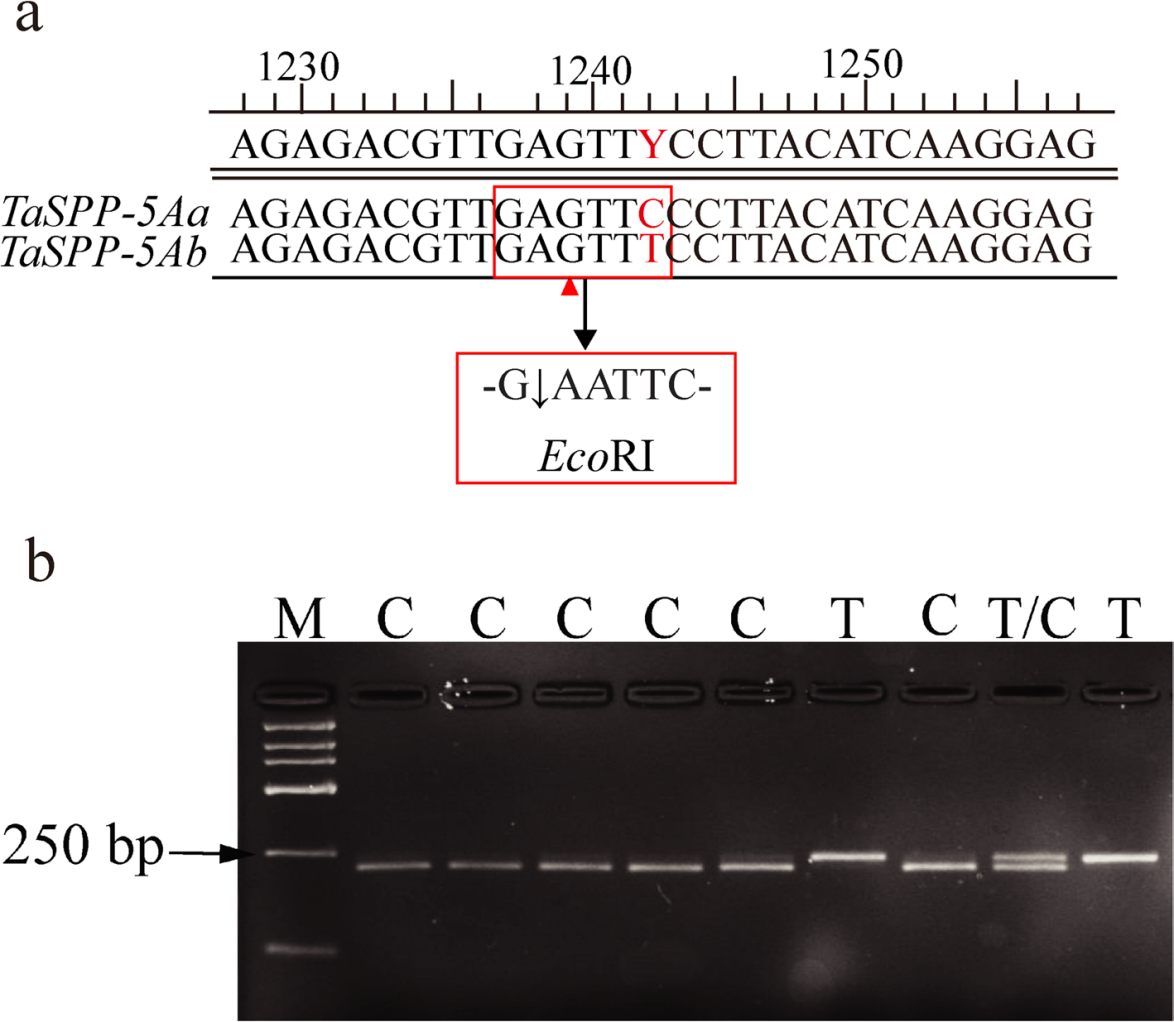
Molecular marker of *TaSPP-5A*. (**a**) Molecular marker *TaSPP-5A-*dCAPS was developed based on the polymorphic SNP (C/T) site. The *Eco*RI restriction site and a base G mismatched to A are marked in red rectangle and red triangle, respectively (**b**) PCR products were digested by *Eco*RI. M is DL2000 DNA marker

**Fig. 3:**
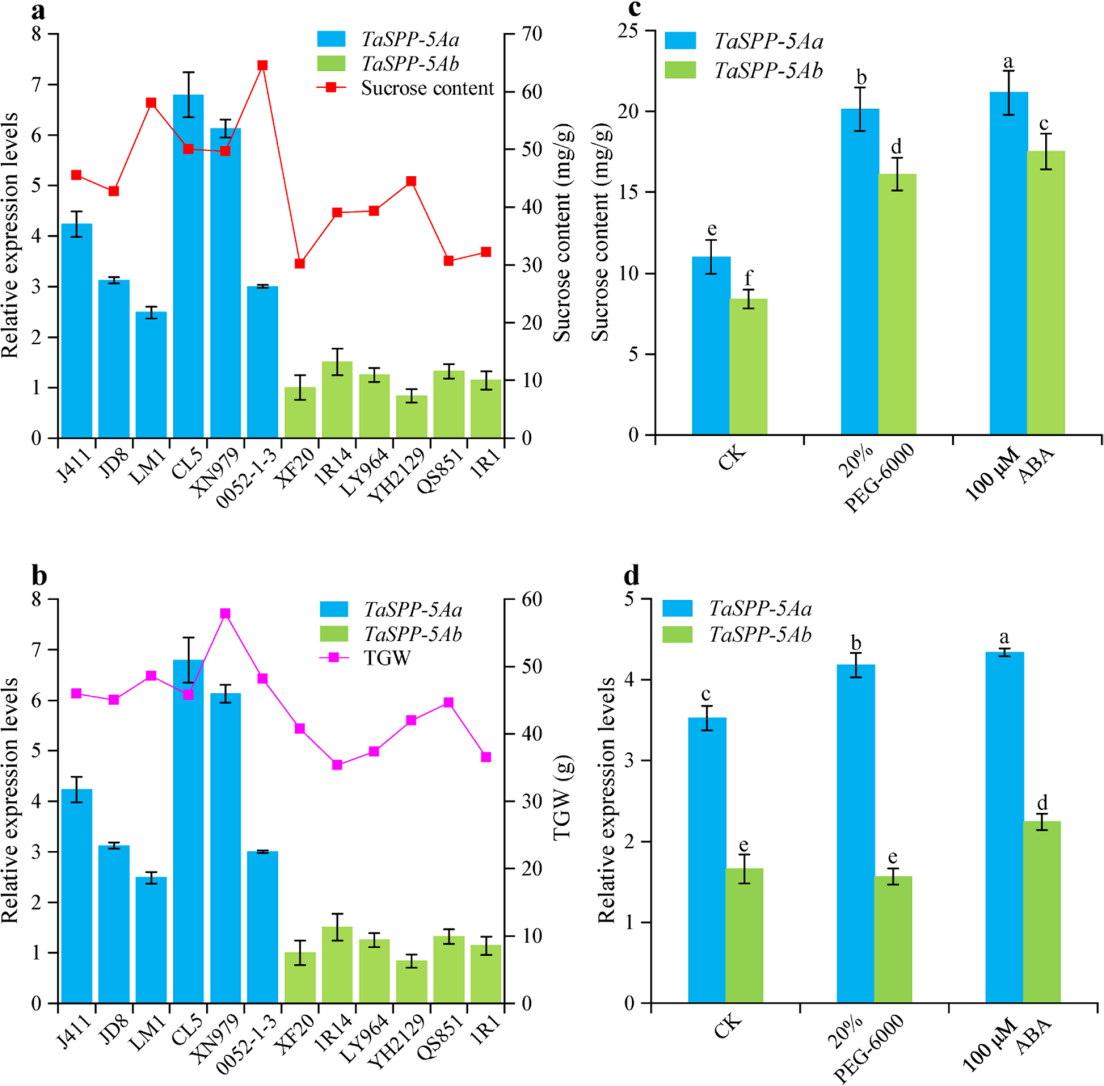
The relative expression levels of *TaSPP-5A* in common wheat varieties carrying *TaSPP-5Aa* or *TaSPP-5Ab* haplotypes were analyzed by qRT-PCR. (**a, b**) Relationship between sucrose content (**a**) and TGW (**b**) with *TaSPP-5A* expression in two *TaSPP-5A* haplotypes. The error bars represent SD from three biological replicates. Each replicate means the data got from a variety plant. XF20 was used as the reference sample. J411: Jing411; JD8: Jingdong8; LM1: Lumai1; CL5: Changle5; XN979: Xinong979; XF20: Xifeng20; LY964: Longyuan964; YH2129: Yunhan2129; QS851: Qingshan851. (**c, d**) Sucrose content (**c**) and expression level **(d**) in two haplotypes at seedling stage under the treatment of 20% PEG-6000 and 100 μM ABA. The error bars represent SD from three biological replicates. Each replicate means the data got from a mixed pool which contained nine different variety plants. The *ACTIN* gene was used as an endogenous control in a, b, and d

Based on the sequence divergence of the allelic variants, a dCAPS marker was developed to distinguish the *TaSPP-5Aa* and *TaSPP-5Ab* haplotypes (Fig. 2; Additional file 1: Table S1). The specific primers were designed to amplify an 843-bp fragment of *TaSPP-5A* containing 1242 site (C-T). A primer pair, *TaSPP-5A*-dCAPS-F and *TaSPP-5A-*dCAPS-R were used to amplify the 235-bp fragment spanning the variation site. The PCR product of 235-bp possessed allelic variation *TaSPP-5Aa* formed an *Eco*RI site GAATTC, whereas the corresponding site in *TaSPP-5Ab* was GAATTT (Fig. 3a). Thus, the 235-bp PCR fragment of *TaSPP-5Aa* was digested into 20-bp and 215-bp parts by restriction enzyme *Eco*RI, whereas the corresponding product from *TaSPP-5Ab* remained 235-bp (Fig. 3b).

### Association analysis between *TaSPP-5A* with sucrose content and grain-related traits

To detect the effects of *TaSPP-5A* allelic variation, the average values of sucrose content, thousand-grain weight (TGW), and grain length (GL) between *TaSPP-5A* allelic variation were compared. A total of 158 varieties were used to validate the dCAPS marker for *TaSPP-5A* gene (Additional file 1: Table S2). A significant association between *TaSPP-5A* and sucrose content was observed in two environments (Table 1). The lines with allele *TaSPP-5Aa* had a significantly higher (*P* < 0.05) sucrose content than those with *TaSPP-5Ab*, suggesting a relationship between *TaSPP-5A* gene and sucrose content in wheat. The association analysis also showed that *TaSPP-5Aa* allelic variation had significantly higher (*P* < 0.05) TGW and GL than those with *TaSPP-5Ab* in three environments (Table 2). Thus, *TaSPP-5Aa* could be a favorable allelic variation for improving grain yield.

**Table 1:** Association analysis of *TaSPP-5A* allelic variation and sucrose content in two environments

Environment	Genotype	No.	Sucrose content Mean±SD	*F*	*P* value
Yuzhong (2014-2015)	*TaSPP-5Aa*	115	19.98±1.10	4.10	0.048^*^
	*TaSPP-5Ab*	43	16.39±0.84		
Tongwei (2018-2019)	*TaSPP-5Aa*	115	38.14±2.21	4.53	0.038^*^
	*TaSPP-5Ab*	43	30.83±2.49		

NO.: Number of accessions; *P < 0.05 and **P < 0.01, respectively

*P* values calculated by the *F* statistics

**Table 2:** Association analysis of *TaSPP-5A* allelic variation and yield-related traits in three environments

Environments	Genotype	No.	TGW Mean±SD	*F*	*P* value	GL Mean±SD	*F*	*P* value
Tongwei (2017-2018)	*TaSPP-5Aa*	115	49.70±0.50	8.53	0.04*	6.92±0.04	4.10	0.045^*^
	*TaSPP-5Ab*	43	46.69±0.93			6.73±0.08		
Tongwei (2018-2019)	*TaSPP-5Aa*	115	42.87±0.82	3.96	0.049*	6.92±0.04	5.32	0.023^*^
	*TaSPP-5Ab*	43	39.56±1.14			6.75±0.05		
Tongwei (2019-2020)	*TaSPP-5Aa*	115	46.22±0.42	11	0.001**	5.98±0.04	4.11	0.045^*^
	*TaSPP-5Ab*	43	43.36±0.79			5.82±0.05		

NO.: Number of accessions; TGW, thousand-grain weight; GL, Grain length; *P < 0.05 and **P < 0.01, respectively

*P* values calculated by the *F* statistics

### *TaSPP-5Aa* haplotype had a higher expression level of *TaSPP-5A* gene compared with *TaSPP-5Ab*

To analyze the effect of favorable allelic variation, 12 varieties possessing *TaSPP-5Aa* or *TaSPP-5Ab* were from 158 varieties were used to test the expression level of *TaSPP-5A* gene. qRT-PCR showed that genotypes with *TaSPP-5Aa* had relatively higher expression levels than those with *TaSPP-5Ab* in grains at the grain-filling stage (Fig. 3a and b), as well as in 14-day-old seedlings (Additional file 1: Figure S3). Consistently, the sucrose content and TGW were generally higher in *TaSPP-5Aa* haplotype than those in *TaSPP-5Ab* (Fig. 3a and b), suggesting that the expression level of *TaSPP-5Aa* might help to maintain high sucrose content and increase wheat yield.

As the metabolism of sucrose has been shown to involve in response to abiotic stress [35, 36], to verify whether *TaSPP-5A* serves any specific roles in the metabolism of sucrose under abiotic stress conditions. Nine wheat varieties with haplotype *TaSPP-5Aa* and nine wheat varieties with haplotype *TaSPP-5Ab* were used to establish two mixed pools under different abiotic stress treatments. Although the sucrose content was significantly induced (*P*<0.05) under both 20% PEG-6000 and 100 μM ABA treatment at two haplotypes (Fig 3c), the expression level of *TaSPP-5A* was slightly or barely induced at haplotypes *TaSPP-5Aa* or haplotypes *TaSPP-5Ab*, respectively (Fig 3d*)*, indicating *TaSPP-5A* may not play a key role under abiotic stress.

### Two *TaSPP-5A* haplotypes comprised 11 SNPs at the promoter region of *TaSPP-5A*, generating putative transcription factor binding sites

**Fig. 4:**
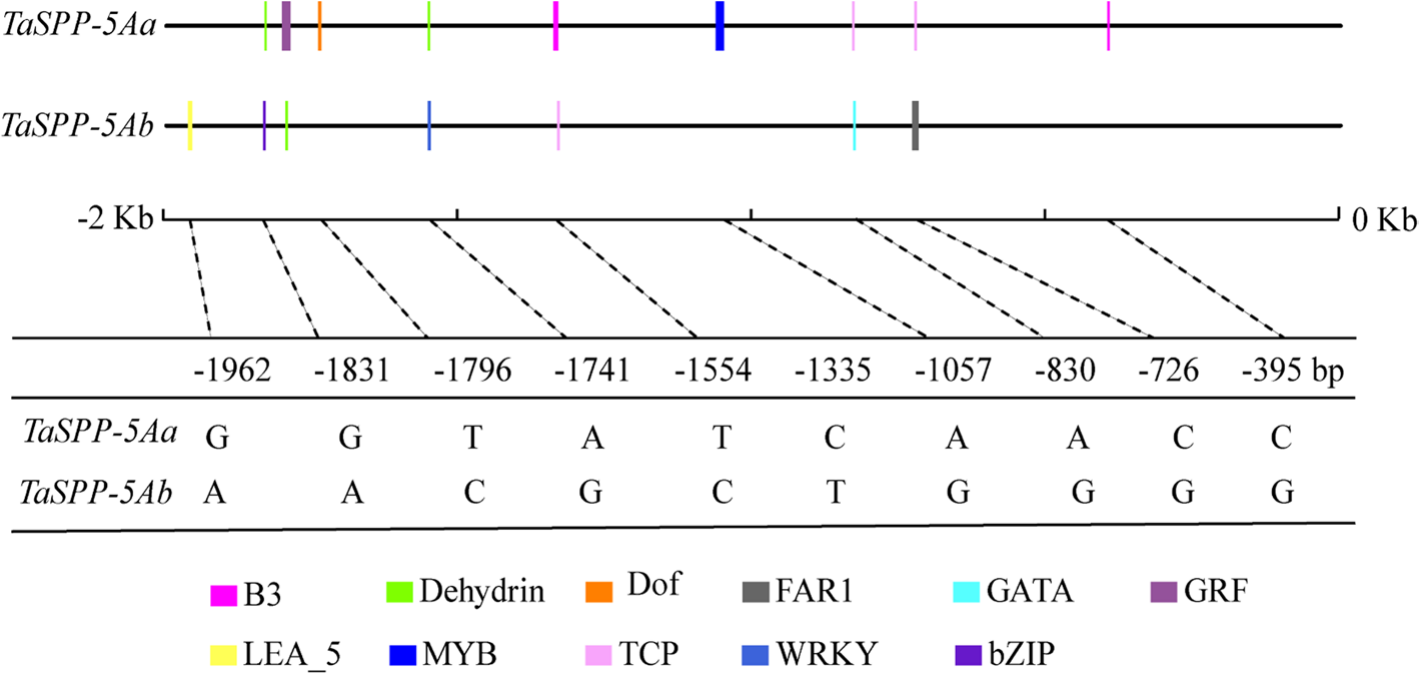
The distribution of main cis-regulatory elements contained SNPs site in the promoter region of the two haplotypes of *TaSPP-5A* gene

As *TaSPP-5Aa* haplotype had higher expression of *TaSPP-5A* gene than *TaSPP-5Ab*, we further detected polymorphisms in the 2,000-bp DNA fragment of *TaSPP-5A* promoter by using 640 varieties from the WheatUnion website (http://wheat.cau.edu.cn/WheatUnion/) and 21 varieties from 158 varieties used for association analysis. A total of 11 SNPs detected in the promoter of *TaSPP-5A* were specifically existed in haplotype *TaSPP-5A* or haplotype *TaSPP-5Ab*, at -395, -726, -830, -1057, -1335, -1554, -1558, -1741, -1796, -1831, and -1962 bp positions, respectively, upstream of the start codon ATG (Fig. 4). Bioinformatic analysis showed that these 11 SNPs participate in the formation of several transcription factor binding sites, such as MYB and B3 binding site (Fig. 4; Additional file 1: Table S3). Therefore, the transcription factor binding site specifically contained in the promoter of *TaSPP-5A* gene in *TaSPP-5Aa* haplotypes may account for the high expression of *TaSPP-5A*.

### *TaSPP-5Aa* underwent selection in Chinese wheat breeding

**Fig. 5:**
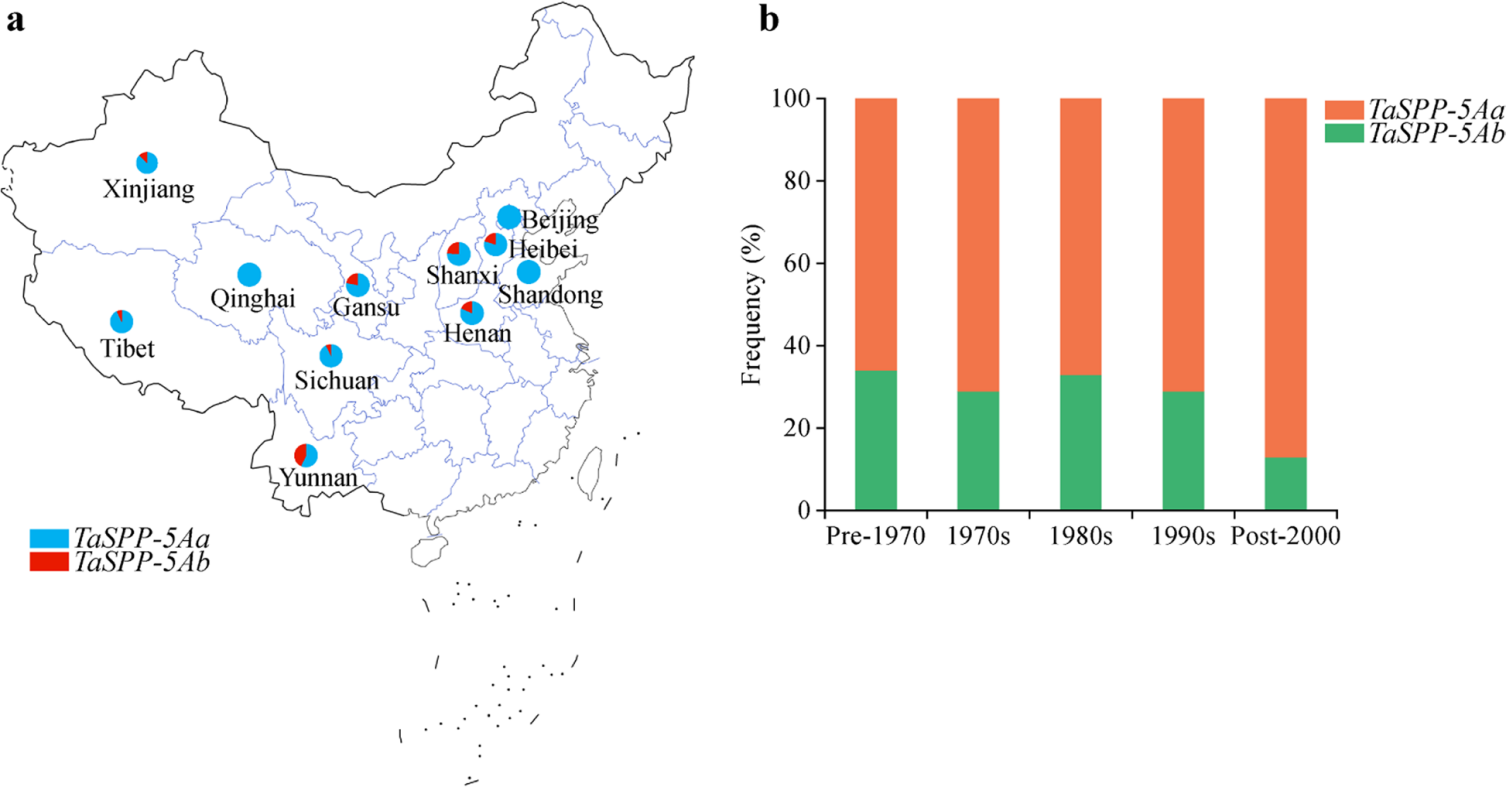
Spatial and temporal distribution of *TaSPP-5A* haplotype. (**a**) Geographic distribution of varieties with *TaSPP-5A* haplotypes in China. The map was downloaded in the Standard Map Service System (http://bzdt.ch.mnr.gov.cn/). (**b**) Frequencies of *TaSPP-5A* allelic variation in Chinese wheat breeding programs in different decades

Artificial selection leaves strong footprints in genomes. As *TaSPP-5Aa* allelic variation had high expression level of *TaSPP-5A* gene, as well as high TGW and GL, we wondered whether the *TaSPP-5Aa* haplotypes were positively selected. Then, the geographic distribution of two *TaSPP-5A* haplotypes was evaluated using 252 cultivated accessions from nine provinces in China (Fig. 5a; Additional file 1: Table S4). The *TaSPP-5Aa* haplotype frequencies from 11 provinces in China were 78% (Gansu), 100% (Beijing), 81.8% (Henan), 80% (Hebei), 100% (Qinghai), 100% (Shandong), 75.7% (Shanxi), 92.9% (Sichuan), 93.2% (Tibet), 87.5% (Xinjiang), and 57.1% (Yunnan). Geographic distribution patterns demonstrated that a favorable *TaSPP-5Aa* haplotype was strongly selected.

To further investigate whether the *TaSPP-5Aa* haplotypes were positively selected during the course of Chinese wheat breeding, the frequencies of *TaSPP-5A* haplotypes in the historical population of 153 accessions were detected in 10-year intervals (pre-1970, 1970s, 1980s, 1990s, and post-2000) (Fig. 5b; Additional file 1: Table S5). The results revealed that *TaSPP-5A*a underwent positive selection, while *TaSPP-5Ab* underwent negative selection, during Chinese wheat breeding process.

## Discussions

### Putative mechanism of *TaSPP-5A* gene in sucrose content

As a product of photosynthesis, sucrose is the main form of carbohydrate translocated from the source to sinks such as seeds and roots [30]. The *SPP* encoding genes have been described in different plant species such as Arabidopsis, tomato, rice, wheat, maize, and coffee [24, 31]. Previous studies have proved that the change of SPP structure can improve the catalytic efficiency and sucrose content [37], and the decreased level of SPP in transgenic tobacco inhibited photosynthesis and altered carbohydrate partitioning with reduced growth rate [22]. The SPP activity repressed in transgenic potato tubers (*Solanum tuberosum*) also results in the decrease of sucrose content [33]. Therefore, *SPP* gene plays a vital role in the regulation of sucrose synthesis. In this study, a 1-bp InDel and three SNPs in the coding region and 11 SNPs in the promoter region of *TaSPP-5A* were identified, exhibiting a significant association with sucrose content.

Sucrose serves as an energy and signaling molecule required for tissue metabolism and as an osmotic to prevent tissue damage in response to abiotic stress [35, 38, 39]. Previous studies have proved that drought stresses increased the sucrose content in leaf and root tissues and phloem sap of rice indica varieties [40]. Several sucrose synthesis- and transport-related genes have been reported to be involved in response to abiotic stress [35, 36, 41]. Sucrose transporters (SUTs) play important roles in the sugar partitioning of source and sink organs. In Arabidopsis, the expression of *AtSUC2* and *AtSUC4* was significantly induced under salt, osmotic, low temperature, and exogenous abscisic acid (ABA) treatments, and the loss-of-function mutation of *AtSUC2* and *AtSUC4* displayed sensitive phenotype responses to abiotic stress and ABA treatment [41]. In Sweet potato, *IbSUT4* also served as a positive regulator in plant stress tolerance through ABA signaling pathway [35]. Sucrose is synthesized by consecutive action of SPS and SPP. The activity of SPS was significantly higher in seedlings of drought-tolerant wheat varieties under normal growth conditions than non-drought-tolerant varieties [36]. The activity of SPS in drought-tolerant wheat varieties was also significantly increased after drought stress treatment [36]. Whether *SPP* genes participate in the regulation of plant abiotic stress tolerance was still unclear. In this study, the average sucrose content was significantly higher in *TaSPP-5Aa* haplotype than those in *TaSPP-5Ab* under both 20% PEG-6000 and 100 μM ABA treatments (Fig. 3d), the rainfall at Tongwei farm station is lower than that at Yuzhong farm station (Supplemental files Fig S4), we speculate that appropriate abiotic is one of the factors that affect the increase in sucrose content, but the expression level of *TaSPP-5A* was slightly or barely induced at haplotypes *TaSPP-5Aa* or haplotypes *TaSPP-5Ab*, respectively. Therefore, these results implied that the *TaSPP-5A* may not play a key role under abiotic stress. Previous researches have shown that the developmental traits were more likely to be controlled by the genes at the A genome, whereas adaptive traits, such as responses to stress and disease, appeared more likely to be controlled by the genes at B and D genomes [42–44]. Therefore, we hypothesized *TaSPP-5A* may mainly serve pivotal roles in the development of wheat grain.

### The transcript regulation of *TaSPP-5A* gene

Transcriptional regulation plays pivotal roles in the activation and suppression of gene expression, and is primarily controlled through gene promoters and their contributing cis-regulatory elements [45]. The expression level of *TaSPP-5A* gene was higher in *TaSPP-5Aa* haplotypes than that in *TaSPP-5Ab* haplotypes, we assumed that the SNPs in *TaSPP-5A* promoter region might affect the expression of *TaSPP-5A*, thus resulting in sucrose content variation in wheat. We used WheatUnion to investigate the diversity of *TaSPP-5A* promoter in 600 wheat varieties, and 11 SNPs were found in the promoter region of *TaSPP-5A*. Analysis of promoter cis-regulatory elements showed that the *TaSPP-5Aa* haplotypes specifically contained several transcription factor binding sites in the promoter region of *TaSPP-5A* gene, such as MYB and B3 transcription factor binding sites, which were not observed in *TaSPP-5Ab* haplotypes (Fig. 5). Previous studies have shown that MYB-type TFs participated in regulating sugar metabolism and the response of multiple abiotic stress. *TaMYB13*, an R2R3-MYB TF, has been characterized as a transcriptional activator of the fructan synthesis pathway in wheat [46]. *CiMYB3* and *CiMYB5* selectively bound the promoter of *1-FEH1*, *1-FEH2a*, and *1-FEH2b* genes to enhance these genes’ expression, without affecting the expression of the fructosyltransferase gene [47]. In potato, MYB TF positively regulated the expression of sucrose hydrolase genes *SUSY1* and *INV2* by directly binding to their promoters [48]. B3, the AFL (ABI3/FUS3/LEC2) subfamily of the B3 TFs, not only alter hormone biosynthesis, mainly involved in ABA and gibberellin, but also regulate the expression of other TFs and/or regulate their downstream activities through protein-protein interactions [49]. Therefore, we assumed that some transcription factors, such as MYB or B3, may specifically bind with the promoter of *TaSPP-5Aa*, but not *TaSPP-5Ab*, and promote the expression of *TaSPP-5Aa*, thus resulting in high content of sucrose in wheat.

### The functional marker *TaSPP-5A-*dCAPS

Functional marker derived from polymorphic sites within genes conferring specific phenotypes has been widely used for marker-assisted selection in wheat breeding, providing a strategy for accelerating the breeding process [50, 51]. Sucrose accumulation- and remobilization-related traits have attracted the attention of breeders for many years, and are generally accepted as determiners of wheat yield. According to the SNPs detected in the *TaSPP-5A* sequence, two haplotypes, *TaSPP-5Aa* and *TaSPP-5Ab*, were identified among wheat accessions. Therefore, we designed a genome-specific primer set to separate orthologous genomic sequences among different wheat varieties and developed a dCAPS marker. Subsequently, their potential value for sucrose content selection was validated by association analysis. In our study, the dCAPS marker *TaSPP-5A*-dCAPS developed from *TaSPP-5A* was associated with sucrose content in flag leaf at the grain-filling stage (Table 1), and we also found that genotypes with favorable allelic variation *TaSPP-5Aa* had higher TGW and GL than *TaSPP-5Ab* allelic variation (Table 2). The functional marker *TaSPP-5A-*dCAPS can be used for marker-assisted selection to improve grain yield, and also for providing a theoretical basis for later research on sucrose accumulation and remobilization in wheat.

## Conclusions

The *TaSPP-5A* gene was cloned. One 1-bp InDel and three SNPs were observed. A molecular marker *TaSPP-5A-*dCAPS was developed based on an SNP at 1242-bp (T-C). Association analysis revealed that favorable allelic variation *TaSPP-5Aa* (C) was associated with high sucrose content, TGW, and GL, compared with *TaSPP-5Ab* allelic variation. qRT-PCR showed the expression of *TaSPP-5A* gene in nine haplotypes, exhibiting a significantly higher expression in *TaSPP-5Aa* than in *TaSPP-5Ab*. The frequency of favorable allelic variation also found that allelic variation *TaSPP-5Aa* underwent strong selection in wheat breeding. Our results provide a functional marker, *TaSPP-5A-*dCAPS, which can be used for marker-assisted selection to improve grain weight in wheat, and also provides insights into the molecular regulation of *TaSPP-5A* gene.

## Materials and methods

### Plant materials

Varieties, including Longxuan987, Xifeng20, Lumai15, and 1R1 with significantly different sucrose contents, were used to clone the full-length sequences of *TaSPP-5A* and identify allelic variations. A total of 158 wheat varieties was used for association analysis of polymorphic markers with agronomic traits (Additional file 1: Table S2), which was provided by Dr. Ruilian Jing at Chinese Academy of Agricultural Sciences [52]. The geographic distribution of two *TaSPP-5A* haplotypes was evaluated using 252 varieties, including 190 varieties accessed from WheatUnion and 62 varieties from 158 varieties used for association analysis (Additional file 1: Table S4). The frequencies of *TaSPP-5A* haplotypes were detected in the historical population of 153 varieties, including 126 varieties accessed from WheatUnion and 27 varieties from 158 varieties used for association analysis (Additional file 1: Table S5). A total of 28 materials were both used for geographic distribution and frequencies analysis. Nine wheat varieties with haplotype *TaSPP-5Aa* and nine wheat varieties with haplotype *TaSPP-5Ab* were used to establish two mixed pools under per stress treatment. Three biological repeats were performed to analyze the data. The seeds were cultivated in a glasshouse at 16/8 h (light/dark, 25/23°C). The varieties were sowed at 9 cm petri dish only contained 13 ml ddH_2_O. The water was poured out after 14 days of growing, and the seedlings were rewatered with 25 ml 100 μM abscisic acid (ABA) and 20% polyethylene glycol-6000 (PEG-6000), then the leaves of seedlings were removed after five days of treatment for the determination of sucrose content and the expression level of *TaSPP-5A*. All wheat accessions were legally obtained from Chinese Crop Germplasm Resources Information System (http://www.cgris.net/zhongzhidinggou/index.php).

### Field trials

The natural population was sown at Yuzhong farm station, Gansu, China (35°48'N, 104°18'E, 1860m ASL) in 2014-2015, and at Tongwei farm Station, Gansu, China (35°110′N, 105°190′E, 1740 m ASL) in 2017-2018, 2018-2019, and 2019-2020. Sucrose content was measured under two environments (2014-2015 and 2018-2019), and TGW and GL were calculated under three environments (2017-2018, 2018-2019, and 2019-2020). Field experimental designs under each environment were randomized complete blocks with three repeats for each variety. Each repeat means the data got from a variety plant. Each plot contained 1 m long with six rows spaced 20 cm apart. Nutrition supplied to all treatments was nitrogen (N) of 180 kg hm^-2^, phosphorus (P_2_O_5_) of 150 kg hm^-2^, and potassium (K_2_O) of 60 kg hm^-2^ only at sowing. Other aspects of field management followed the local practices in wheat production. All the fresh samples were first dried at 100 °C for 30 min and thereafter dried at 80 °C until constant dry weight. The dehydrated samples were chipped into pieces of 3-5 mm in length. Subsequently sucrose content was measured using the Resorcin method as described by Yemm et al. [53]. Using a Microplate Reader scanned wavelengths at 500 nm reflectance mode. Agronomic traits have been measured by the SC-G2 kernel testing equipment developed by Wanshen Science and Technology Ltd. (Hangzhou, China)

### Cloning and sequence analysis of *TaSPP-5A*

The rice gene *OsSPP* (GenBank accession: AA752973) was used as the query for BLAST search against the *Ensembl*Plant (http://plants.ensembl.org/index.html). Based on the sequence alignment, a specific primer pair of *TaSPP-5A*-F1/R1 was designed to amplify wheat *SPP-5A* gene (Additional file 1: Table S1). Genomic DNA from fresh leaves of 14-day-old wheat seedlings was extracted by CTAB method [54]. PCR was performed in a total volume of 20 μL, including 2 pM of each primer, 1.6 μL of 2.5 mM dNTPs, 150 ng of template DNA, 4 μL of 5×TransStart *FastPfu* buffer, and 0.4 μL of 2.5 U TransStart *FastPfu* DNA Polymerase (TransGen Biotech, Beijing). The PCR conditions were an initial denaturation at 95°C for 5 min, followed by 35 cycles of 95°C for 1 min, annealing 57°C for 20 s, and extension at 72°C for 1.5 min, with a final extension of 72°C for 10 min. The PCR products were separated by 1% agarose gels electrophoresis and the target bands recovered, cloned into the pEASY-Blunt Cloning vector (TransGen Biotech, Beijing), and sequenced by Shanghai Sangon Biotech Co. Ltd (http://www.sangon.com). The genomic DNA sequences of the *TaSPP-5A* were aligned using the SeqMAN and MegAlign software (DNASTAR Lasergene 6.1.0) to detect allelic variants among accessions.

### Polymorphism identification and development of a functional marker

Based on the SNP polymorphism sites (1242 bp-C/T), the specific primers (Additional file 1: Table S1) were designed to amplify *TaSPP* and a dCAPS was developed. By two rounds of PCR, the sequence of *TaSPP-5A* at 1242 sites (C-T) was amplified by *TaSPP-5A*-F2/R2, then the first round PCR products were diluted 200 times, and the second round PCR was performed with the primer pair *TaSPP-5A-*dCAPS-F/R using 1 μL diluted PCR products as template. Finally, PCR products were digested with the restriction enzyme *Eco*RI (Sangon Biotech, Shanghai) and the digested PCR products were separated using 5% agarose gels.

### RNA extraction and quantitative Real-Time PCR

Total RNA samples were extracted using an E.Z.N.A.®Plant RNA Kit (Omega, Shanghai). First-strand cDNA was synthesized with Rever Tra Ace qPCR RT Master Mix with gDNA Remover (TOYOBO, Japan). Quantitative real-time PCR (qRT-PCR) was performed for gene expression studies. Primer sequences used to amplify *TaSPP-5A* and *TaACTIN* (control) genes were shown in Table S1. qPT-PCR was performed with Roche LightCycler® 96 (Roche, Switzerland) using the KOD SYBR qPCR Mix (TOYOBO, Japan). The reaction procedure was as follows: thermal cycling at 98°C for 120 s, followed by 40 cycles at 98°C for 10 s, 60°C for 10 s, and 68°C for 30 s. Three biological repeats were used for qRT-PCR analysis. Each repeat means the data got from a variety plant. The relative transcript level of *TaSPP-5Aa* and *TaSPP-5Ab* was determined using the 2^-ΔΔCt^ method [55].

### Statistical analysis

One-way analysis of variance (ANOVA) was used to determine the significance between different haplotypes and phenotypic traits in SPSS19.0 software ( http://www.ibm.com ).

### Analysis of the cis-regulatory elements in the promoter

The sequences of the 2,000-bp promoter in *TaSPP-5A* genes were retrieved from the *Ensembl*Plants database (http://plants.ensembl.org/index.html). Wheat genome variation consortium database (http://wheat.cau.edu.cn/WheatUnion/) was applied to obtain variant loci in promoters of different materials [56–58]. PlantPAN 3.0 (http://PlantPAN.itps.ncku.edu.tw/) was used to analyze the cis-regulatory elements in the promoter of *TaSPP-5A*. The distribution of identified cis-regulatory elements was drawn by the GSDS (http://gsds.gao-lab.org/).

## Supplementary Information

**Additional file 1: Figure S1.** The PCR amplification of *TaSPP-5A* gene in different wheat varieties. **Figure S2.** Alignment of the cloned *TaSPP-5A* orthologs. The restriction site *Eco*RI and the locations of primers *TaSPP-5A*-F2/R2 and *TaSPP-5A-*dCAPS-F/R were labeled by lines with arrow. **Figure S3.** The expression level of *TaSPP-5A* and the content of sucrose in 14-day-old wheat seedlings carrying *TaSPP-5Aa* or *TaSPP-5Ab* haplotypes. **Figure S4.** The rainfall for each growing season in the two tested environments. **Table S1.** Primer sequences used in this study. **Table S2.** The information of the wheat diversity panel and their genotypes of *TaSPP-5A* alleles. **Table S3.** Cis-regulatory elements contained SNPs site in the promoter region of the two haplotypes of *TaSPP-5A* gene and their sequences**. Table S4.** The information of the wheat diversity panel and geographic distribution of *TaSPP-5A* alleles. **Table S5.** The information of the wheat diversity panel and their genotypes of *TaSPP-5A* alleles in the different decades

## Data Availability

All data generated or analyzed during this study are included in this published article and its supplementary information files. The datasets used and/or analyzed during the current study are available from the corresponding author on reasonable request. The sequencing data has been submitted to the NCBI SRA database (BioProject ID PRJNA762528, https://www.ncbi.nlm.nih.gov/sra/PRJNA762528).

## Abbreviations

SPP: Sucrose phosphatase
dCAPS: Derived cleaved amplified polymorphic sequence
SNP: Single nucleotide polymorphism
Indel: Insertion and Deletion
TGW: Thousand-grain weight
GL: Grain length
