# Supplementary material for: Characterization of TaSPP-5A gene associated with sucrose content in wheat (Triticum aestivum L.)
Source: BMC Plant Biol. 2022 Feb 1;22:58. doi: 10.1186/s12870-022-03442-x (PMC8805233; doi:10.1186/s12870-022-03442-x)
Supplement: Supplementary file 1 — Additional file 1: Figure S1. The PCR amplification of TaSPP-5A gene in different wheat varieties. Figure S2. Alignment of the cloned TaSPP-5A orthologs. The restriction site EcoRI and the locations of primers TaSPP-5A-F2/R2 and TaSPP-5A-dCAPS-F/R were labeled by lines with arrow. Figure S3. The expression level of TaSPP-5A and the content of sucrose in 14-day-old wheat seedlings carrying TaSPP-5Aa or TaSPP-5Ab haplotypes. Figure S4. The rainfall for each growing season in the two tested environments. Table S1. Primer sequences used in this study. Table S2. The information of the wheat diversity panel and their genotypes of TaSPP-5A alleles. Table S3. Cis-regulatory elements contained SNPs site in the promoter region of the two haplotypes of TaSPP-5A gene and their sequences. Table S4. The information of the wheat diversity panel and geographic distribution of TaSPP-5A alleles. Table S5. The information of the wheat diversity panel and their genotypes of TaSPP-5A alleles in the different decades [file 12870_2022_3442_MOESM1_ESM.docx]

**SUPPLEMENTAL MATERIALS
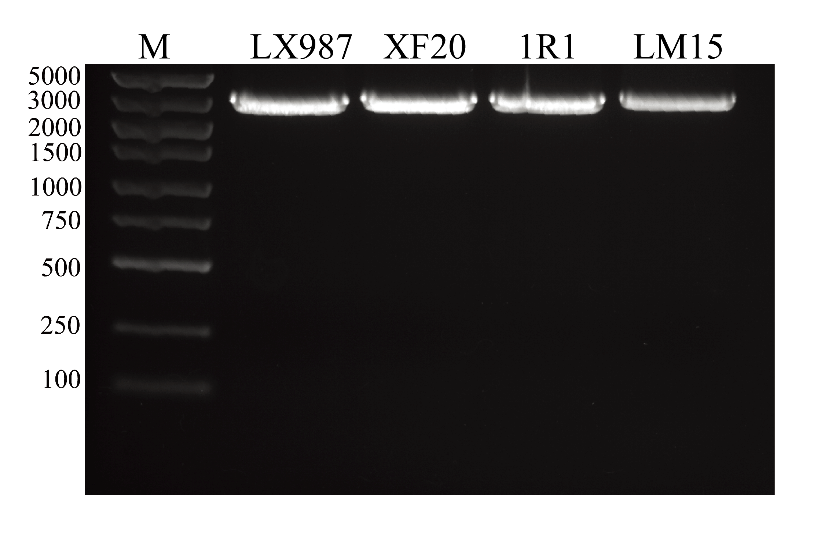
**

**Figure S1.** The PCR amplification of *TaSPP-5A* gene in different wheat varieties. M: Marker; LX987: Lunxuan987; XF20: Xifeng20; LM15: Lumai15


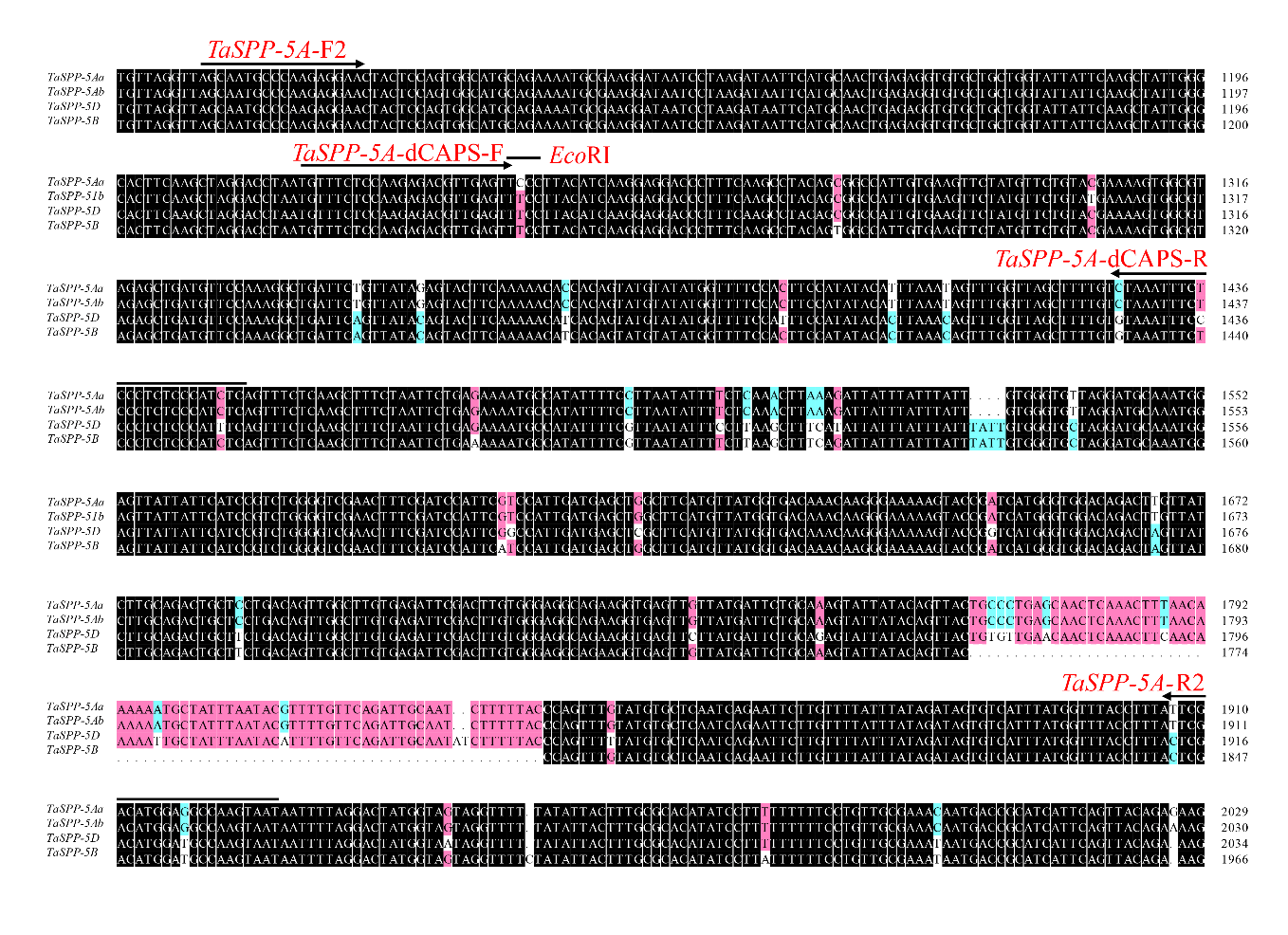


**Figure S2.** Alignment of the cloned *TaSPP-5A* orthologs. The restriction site *Eco*RⅠ and the locations of primers *TaSPP-5A*-F2/R2 and *TaSPP-5A-*dCAPS-F/R were labeled by lines with arrow


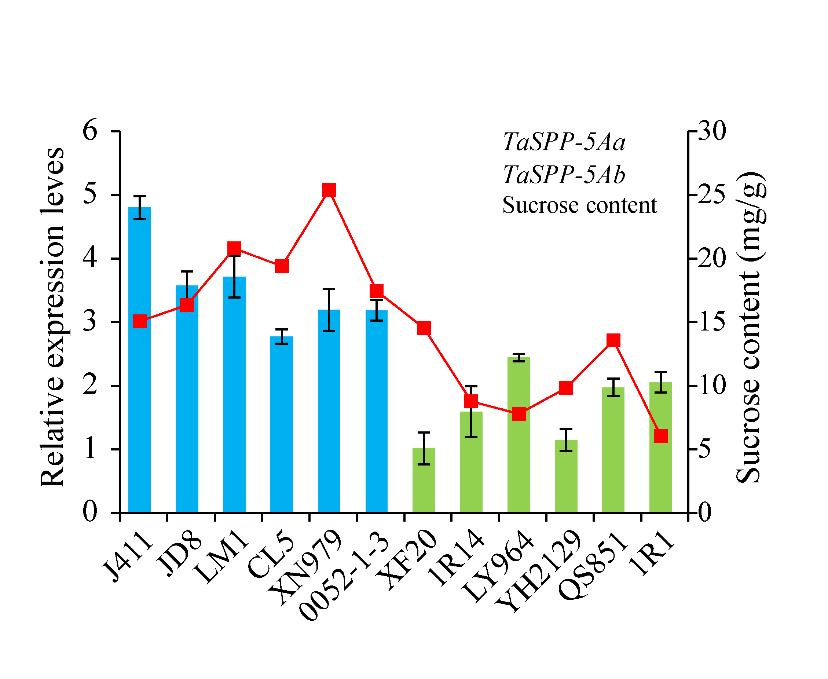


**Figure S3.** The expression level of *TaSPP-5A* and the content of sucrose in 14-day-old seedlings carrying *TaSPP-5Aa* or *TaSPP-5Ab* haplotypes.


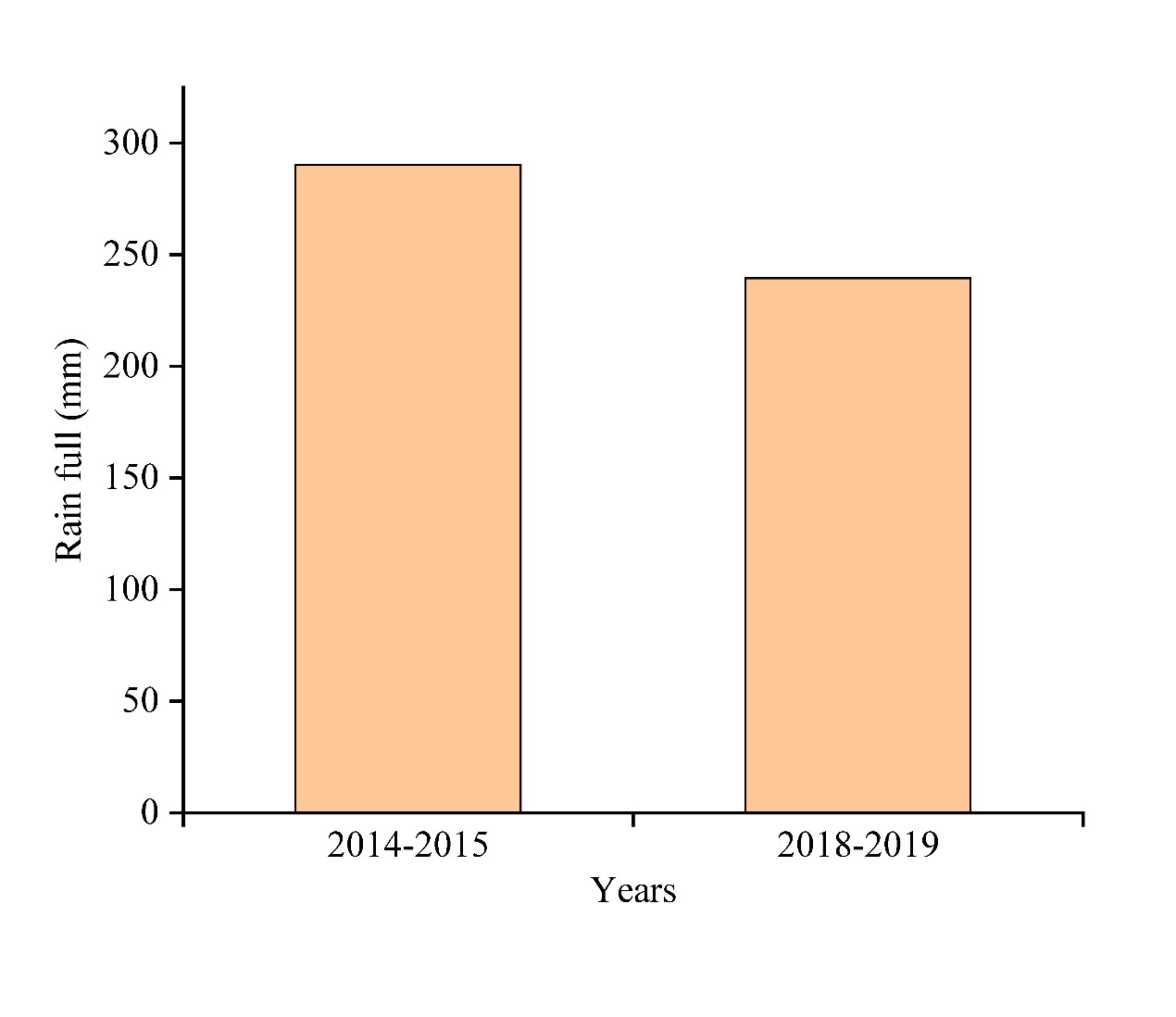


**Figure S4.** The rainfall for each growing season in the two tested environments.

**Table S1.** Primer sequences used in this study

| Primer set | Primer sequence (5ˊ-3ˊ) |
| --- | --- |
| *TaSPP-5A*-F1 | TGACGAACCCTAACCTGCTT |
| *TaSPP-5A-*R1 | ATCAAGAGACCTCCGTAGACAT |
| *TaSPP-5B-F1* | CTGCCTGTGAAAGTCTGGGTT |
| *TaSPP-5B-R1* | TGGGACTGGGGTCATTTACTTT |
| *TaSPP-5D-F1* | CGAATCTTAATTTCTCACAGAGG |
| *TaSPP-5D-R1* | ATCCAGTCCAGTATGTGTGTCT |
| *TaSPP-5A*-F2 | AGCAATGCCCAAGAGGAA |
| *TaSPP-5A*-R2 | ATTACTTGGCCTCCATGTCGAA |
| *TaSPP-5A*-dCAPS-F1 | TGTTTCTCCAAGAGACGTTAATT |
| *TaSPP-5A*-dCAPS-R1 | TGAGATGGGAGAGGGAGAAATTTAG |
| *TaSPP-5A-F3* | GGAAGTGGGCAGTGGTTCAC |
| *TaSPP-5A-R3* | CTGCGAGTTTAGACAAACACCATT |
| *TaSPP-5A-*F | CTTGCAGACTGCTCCTGACA |
| *TaSPP-5A-*R | TTTGGATGACTGCTCGACCC |
| *TaACTIN-*F | GACCCAGACAACTCGCAAC |
| *TaACTIN-*R | GGAATCCATGACCACCTAC |

**Table S2****.** The information of the wheat diversity panel and their genotypes of *TaSPP-5A*

| Number | Accession | Allele | Number | Accession | Allele |
| --- | --- | --- | --- | --- | --- |
| 1 | Lantian10 | C | 80 | Beijing8686 | C |
| 2 | Lantian11 | C | 81 | Changle5 | C |
| 3 | Lantian12 | C | 82 | Chang4640 | C |
| 4 | Lantian14 | C | 83 | Chang4738 | C |
| 5 | Lantian16 | C | 84 | Chang6154 | C |
| 6 | Lantian18 | C | 85 | Chang6359 | C |
| 7 | Lantian19 | C | 86 | Chang8744 | C |
| 8 | Lantian2 | C | 87 | Han4589 | C |
| 9 | Lantian20 | C | 88 | Hanxuan10 | C |
| 10 | Lantian21 | C | 89 | Jimai21 | C |
| 11 | Lantian22 | C | 90 | Jimai32 | C |
| 12 | Lantian23 | C | 91 | Jinnong207 | C |
| 13 | Lantian24 | C | 92 | Keyi29 | C |
| 14 | Lantian25 | C | 93 | Longjian196 | C |
| 15 | Lantian26 | C | 94 | Longjian294 | C |
| 16 | Lantian27 | C | 95 | Lumai1 | C |
| 17 | Lantian28 | C | 96 | Chang4378 | C |
| 18 | Lantian29 | C | 97 | Lumai15 | C |
| 19 | Lantian3 | C | 98 | Lunxuan987 | C |
| 20 | Lantian30 | C | 99 | Shi4185 | C |
| 21 | Lantian33 | C | 100 | Xinong688 | C |
| 22 | Lantian34 | C | 101 | Xinong797 | C |
| 23 | Lantian35 | C | 102 | Xinong979 | C |
| 24 | Lantian36 | C | 103 | Longjian101 | C |
| 25 | Lantian4 | C | 104 | Longjian107 | C |
| 26 | Lantian5 | C | 105 | Chang7080 | C |
| 27 | Lantian6 | C | 106 | Jimai262 | C |
| 28 | Lantian7 | C | 107 | Jimai22 | C |
| 29 | Zhongmai175 | C | 108 | Jimai19 | C |
| 30 | Zhongyou9507 | C | 109 | Jimai20 | C |
| 31 | Lantian8 | C | 110 | Jimai229 | C |
| 32 | Lantian9 | C | 111 | Jimai23 | C |
| 33 | Longjian108 | C | 112 | Jimai44 | C |
| 34 | Longjian110 | C | 113 | Chang6794 | C |
| 35 | Longjian111 | C | 114 | Chang6135 | C |
| 36 | C28-5-1-3 | C | 115 | Nongda311 | C |
| 37 | C72-1-3-2-2 | C | 116 | Jinmai47 | T |
| 38 | E72-2-2 | C | 117 | Jinmai79 | T |
| 39 | Hangxuan01 | C | 118 | Jinmai68 | T |
| 40 | Hangxuan121 | C | 119 | Jinmai63 | T |
| 41 | Chang721 | C | 120 | Longyuan964 | T |
| 42 | Chang844 | C | 121 | Longzimai1 | T |
| 43 | Chang9325 | C | 122 | Jinmai72 | T |
| 44 | Lantian13 | C | 123 | Pubing151 | T |
| 45 | Lantian15 | C | 124 | Yumai18 | T |
| 46 | A88-4-2-4 | C | 125 | 1R8 | T |
| 47 | Chang4758 | C | 126 | 1R14 | T |
| 48 | 29-2-2 | C | 127 | 1R20 | T |
| 49 | Longmai847 | C | 128 | 1R19 | T |
| 50 | Longjian4 | C | 129 | 0052-1-4-1 | T |
| 51 | Longjian104 | C | 130 | 1R6 | T |
| 52 | Longyu5 | C | 131 | 1R5 | T |
| 53 | Longjian103 | C | 132 | 1R1 | T |
| 54 | Longjian169 | C | 133 | 1R39 | T |
| 55 | Chang6878 | C | 134 | 1R38 | T |
| 56 | Lumai14 | C | 135 | 1R27 | T |
| 57 | Jingdong8 | C | 136 | 1R25 | T |
| 58 | Yunhan2028 | C | 137 | 1R26 | T |
| 59 | Jing411 | C | 138 | 1R2 | T |
| 60 | Longzhong2 | C | 139 | 1R17 | T |
| 61 | Chang6452 | C | 140 | 1R11 | T |
| 62 | Ningmai5 | C | 141 | Yunhan2129 | T |
| 63 | Q9086 | C | 142 | Cangmai6001 | T |
| 64 | 9840-0-3-2 | C | 143 | Heng7228 | T |
| 65 | Chang6738 | C | 144 | Qingshan843 | T |
| 66 | Longjian127 | C | 145 | Qingshan851 | T |
| 67 | Longjian3 | C | 146 | Xifeng27 | T |
| 68 | Qingnong3 | C | 147 | Xifeng28 | T |
| 69 | 0052-1-3 | C | 148 | Xiannong4 | T |
| 70 | Qingnong4 | C | 149 | Lude1 | T |
| 71 | Longjian301 | C | 150 | Shijiazhuang8 | T |
| 72 | Longjian386 | C | 151 | Xifeng16 | T |
| 73 | 94164-1 | C | 152 | Xifeng20 | T |
| 74 | Longjian387 | C | 153 | Xifeng18 | T |
| 75 | Longjian385 | C | 154 | Jinmai73 | T |
| 76 | Linhan234 | C | 155 | Xifeng24 | T |
| 77 | Linhan538 | C | 156 | Xifeng19 | T |
| 78 | Linhan51241 | C | 157 | Jinmai40 | T |
| 79 | Baiqimai | C | 158 | Jinmai8 | T |

**Table S3.** Cis-regulatory elements contained SNPs site in the promoter region of the two haplotypes of *TaSPP-5A* gene and their sequences

| Site (bp) | *TaSPP-5Aa* | Sequences | *TaSPP-5Ab* | Sequences |
| --- | --- | --- | --- | --- |
| -395 | B3 | CCTGC |  |  |
| -726 | TCP, Dof | GGCGC, AAGGC | FAR1 | GAAACGCGCTCG |
| -830 | TCP | GGACA | GATA | AGATG |
| -1057 | MYB | GTGTTCTAACATTTT |  |  |
| -1335 | B3 | CGCGACAGC | TCP | GGCGC |
| -1554 | Dehydrin | TCGAC | WRKY | TTGACG |
| -1558 |  |  |  |  |
| -1741 | Dof | AAAGC |  |  |
| -1796 | GRF | CTGTCAGGTGGCACC | Dehydrin | CCGTC |
| -1831 | Dehydrin | CCGCC | bZIP | CGCCA |
| -1962 |  |  | LEA_5 | CATGCACG |

SNPs site were marked in red

**Table S4.** The information of the wheat diversity panel and their genotypes distribution of *TaSPP-5A* alleles

| Number | Accession | Nation | Origin | Allele | Source |
| --- | --- | --- | --- | --- | --- |
| 1 | DF_BJYanDa1817 | China | Beijing | C | WheatUnion |
| 2 | YC_NongDa3338 | China | Beijing | C | WheatUnion |
| 3 | YC_NongDa5181 | China | Beijing | C | WheatUnion |
| 4 | YCL_Lovrin10 | China | Beijing | C | WheatUnion |
| 5 | YCL_BeiJing8 | China | Beijing | C | WheatUnion |
| 6 | YC_LX987 | China | Beijing | C | WheatUnion |
| 7 | YC_Jing411 | China | Beijing | C | WheatUnion |
| 8 | YC_NongDa3097 | China | Beijing | C | WheatUnion |
| 9 | YC_NongDa3331 | China | Beijing | C | WheatUnion |
| 10 | YC_JingDong6 | China | Beijing | C | WheatUnion |
| 11 | Lantian10 | China | Gansu | C | Laboratory |
| 12 | Lantian11 | China | Gansu | C | Laboratory |
| 13 | Lantian12 | China | Gansu | C | Laboratory |
| 14 | Lantian14 | China | Gansu | C | Laboratory |
| 15 | Lantian16 | China | Gansu | C | Laboratory |
| 16 | Lantian18 | China | Gansu | C | Laboratory |
| 17 | Lantian19 | China | Gansu | C | Laboratory |
| 18 | Lantian2 | China | Gansu | C | Laboratory |
| 19 | Lantian20 | China | Gansu | C | Laboratory |
| 20 | Lantian21 | China | Gansu | C | Laboratory |
| 21 | Lantian22 | China | Gansu | C | Laboratory |
| 22 | Lantian23 | China | Gansu | C | Laboratory |
| 23 | Lantian24 | China | Gansu | C | Laboratory |
| 24 | Lantian25 | China | Gansu | C | Laboratory |
| 25 | Lantian26 | China | Gansu | C | Laboratory |
| 26 | Lantian27 | China | Gansu | C | Laboratory |
| 27 | Lantian28 | China | Gansu | C | Laboratory |
| 28 | Lantian29 | China | Gansu | C | Laboratory |
| 29 | Lantian3 | China | Gansu | C | Laboratory |
| 30 | Lantian30 | China | Gansu | C | Laboratory |
| 31 | Lantian33 | China | Gansu | C | Laboratory |
| 32 | Lantian34 | China | Gansu | C | Laboratory |
| 33 | Lantian35 | China | Gansu | C | Laboratory |
| 34 | Lantian36 | China | Gansu | C | Laboratory |
| 35 | Lantian4 | China | Gansu | C | Laboratory |
| 36 | Lantian5 | China | Gansu | C | Laboratory |
| 37 | Lantian6 | China | Gansu | C | Laboratory |
| 38 | Lantian7 | China | Gansu | C | Laboratory |
| 39 | Xifeng27 | China | Gansu | T | Laboratory |
| 40 | Xifeng28 | China | Gansu | T | Laboratory |
| 41 | Xifeng16 | China | Gansu | T | Laboratory |
| 42 | Xifeng20 | China | Gansu | T | Laboratory |
| 43 | Xifeng18 | China | Gansu | T | Laboratory |
| 44 | Qingshan843 | China | Gansu | T | Laboratory |
| 45 | DF_GSHongQiMai | China | Gansu | T | WheatUnion |
| 46 | DF_GSDuanYaoMai | China | Gansu | C | WheatUnion |
| 47 | DF_GSDaBaiMai | China | Gansu | T | WheatUnion |
| 48 | DF_GSBaiMuXianBan | China | Gansu | T | WheatUnion |
| 49 | DF_GSBaiDaTou | China | Gansu | C | WheatUnion |
| 50 | DF_GSLanHuaMai | China | Gansu | C | WheatUnion |
| 51 | DF_GSYiZhiMai | China | Gansu | C | WheatUnion |
| 52 | BS_HNPuYang21 | China | Henan | C | WheatUnion |
| 53 | BS_HNPuYang23 | China | Henan | C | WheatUnion |
| 54 | YC_ZhouMai18 | China | Henan | C | WheatUnion |
| 55 | YC_LuMai21 | China | Henan | C | WheatUnion |
| 56 | YC_YuMai18 | China | Henan | T | WheatUnion |
| 57 | YCL_ZhengMai9023 | China | Henan | C | WheatUnion |
| 58 | YCL_YanZhan1 | China | Henan | C | WheatUnion |
| 59 | YCL_YuMai21 | China | Henan | T | WheatUnion |
| 60 | YCL_ZhengYin4 | China | Henan | C | WheatUnion |
| 61 | YCL_BaiNong3217 | China | Henan | C | WheatUnion |
| 62 | VA_China2 | China | Henan | C | WheatUnion |
| 63 | DF_QHXiaoHongMai | China | Qinghai | C | WheatUnion |
| 64 | DF_QHXiaoMai | China | Qinghai | C | WheatUnion |
| 65 | DF_QHMaoHongMai | China | Qinghai | C | WheatUnion |
| 66 | DF_QHLiuYueHuang | China | Qinghai | C | WheatUnion |
| 67 | DF_QHDuanBaiMai | China | Qinghai | C | WheatUnion |
| 68 | DF_QHGeJiaXiang | China | Qinghai | C | WheatUnion |
| 69 | BS_SDNingYang5 | China | Shandong | C | WheatUnion |
| 70 | BS_SDChangQing49 | China | Shandong | C | WheatUnion |
| 71 | DF_SDXiShanBianSui | China | Shandong | C | WheatUnion |
| 72 | DF_SDBaiBianSui | China | Shandong | C | WheatUnion |
| 73 | DF_SDDaLiBanMang | China | Shandong | C | WheatUnion |
| 74 | DF_SDLaoLaiXia | China | Shandong | C | WheatUnion |
| 75 | YCL_JiNan17 | China | Shandong | C | WheatUnion |
| 76 | YCL_TaiShan1 | China | Shandong | C | WheatUnion |
| 77 | YC_JiMai22 | China | Shandong | C | WheatUnion |
| 78 | VA_China4 | China | Shandong | C | WheatUnion |
| 79 | Jinmai47 | China | Shanxi | T | Laboratory |
| 80 | Jinmai79 | China | Shanxi | T | Laboratory |
| 81 | Jinmai68 | China | Shanxi | T | Laboratory |
| 82 | Jinmai63 | China | Shanxi | T | Laboratory |
| 83 | Jinmai72 | China | Shanxi | T | Laboratory |
| 84 | Jinmai73 | China | Shanxi | T | Laboratory |
| 85 | Jinmai40 | China | Shanxi | T | Laboratory |
| 86 | Jinmai8 | China | Shanxi | T | Laboratory |
| 87 | Linhan538 | China | Shanxi | C | Laboratory |
| 88 | Linhan51241 | China | Shanxi | C | Laboratory |
| 89 | Linhan234 | China | Shanxi | C | Laboratory |
| 90 | Chang721 | China | Shanxi | C | Laboratory |
| 91 | Chang844 | China | Shanxi | C | Laboratory |
| 92 | Chang9325 | China | Shanxi | C | Laboratory |
| 93 | Chang4758 | China | Shanxi | C | Laboratory |
| 94 | Chang6878 | China | Shanxi | C | Laboratory |
| 95 | Chang6452 | China | Shanxi | C | Laboratory |
| 96 | Chang6738 | China | Shanxi | C | Laboratory |
| 97 | Chang4640 | China | Shanxi | C | Laboratory |
| 98 | Chang4738 | China | Shanxi | C | Laboratory |
| 99 | Chang6154 | China | Shanxi | C | Laboratory |
| 100 | Chang6359 | China | Shanxi | C | Laboratory |
| 101 | Chang8744 | China | Shanxi | C | Laboratory |
| 102 | Chang4378 | China | Shanxi | C | Laboratory |
| 103 | Chang7080 | China | Shanxi | C | Laboratory |
| 104 | Chang6794 | China | Shanxi | C | Laboratory |
| 105 | Chang6135 | China | Shanxi | C | Laboratory |
| 106 | DF_SXDingXingZhai | China | Shanxi | T | WheatUnion |
| 107 | DF_SXBaiHuoMai | China | Shanxi | C | WheatUnion |
| 108 | DF_SHXChunXiaoMai | China | Shanxi | C | WheatUnion |
| 109 | DF_SHXMaZhaMai | China | Shanxi | C | WheatUnion |
| 110 | DF_SHXXiaoSanYueH | China | Shanxi | C | WheatUnion |
| 111 | YCL_ChangZhi6406 | China | Shanxi | C | WheatUnion |
| 112 | YCL_BiMa4 | China | Shanxi | C | WheatUnion |
| 113 | YCL_FengChan3 | China | Shanxi | C | WheatUnion |
| 114 | VA_China3 | China | Shanxi | C | WheatUnion |
| 115 | VA_China7 | China | Shanxi | C | WheatUnion |
| 116 | DF_SC1658 | China | Shanxi | C | WheatUnion |
| 117 | DF_SC1666 | China | Shanxi | C | WheatUnion |
| 118 | DF_SC1587 | China | Sichuan | C | WheatUnion |
| 119 | DF_SC1635 | China | Sichuan | C | WheatUnion |
| 120 | DF_SC1588 | China | Sichuan | C | WheatUnion |
| 121 | DF_SC1668 | China | Sichuan | T | WheatUnion |
| 122 | DF_SC1661 | China | Sichuan | C | WheatUnion |
| 123 | DF_SC1670 | China | Sichuan | C | WheatUnion |
| 124 | YCL_Fan6 | China | Sichuan | C | WheatUnion |
| 125 | DF_SCBenDiHuangHua | China | Sichuan | C | WheatUnion |
| 126 | DF_SCBaiMangXiaoMai | China | Sichuan | C | WheatUnion |
| 127 | DF_SCKangDing1 | China | Sichuan | C | WheatUnion |
| 128 | LR_China3 | China | Sichuan | C | WheatUnion |
| 129 | VA_China5 | China | Sichuan | C | WheatUnion |
| 130 | XZbys_Zang1817 | China | Tibet | C | WheatUnion |
| 131 | XZbys_ZuoGong1203 | China | Tibet | C | WheatUnion |
| 132 | XZbys_LangXian1274 | China | Tibet | C | WheatUnion |
| 133 | XZbys_JiTang1387 | China | Tibet | C | WheatUnion |
| 134 | XZMiSui | China | Tibet | C | WheatUnion |
| 135 | XZD_ZXM1341 | China | Tibet | T | WheatUnion |
| 136 | XZbys_ZuoGongSuiSui1 | China | Tibet | C | WheatUnion |
| 137 | XZbys_JiaChaSuiSui8 | China | Tibet | C | WheatUnion |
| 138 | XZbys_JiaChaSuiSui27 | China | Tibet | C | WheatUnion |
| 139 | XZbys_JiaChaSuiSui32 | China | Tibet | C | WheatUnion |
| 140 | XZbys_JiaChaSuiSui33 | China | Tibet | T | WheatUnion |
| 141 | XZbys_JiaChaSuiSui34 | China | Tibet | C | WheatUnion |
| 142 | XZbys_LangXianSuiSui9 | China | Tibet | C | WheatUnion |
| 143 | XZbys_LangXianSuiSui15 | China | Tibet | C | WheatUnion |
| 144 | XZbys_LangXianSuiSui17 | China | Tibet | C | WheatUnion |
| 145 | XZbys_LongZiSuiSui10 | China | Tibet | C | WheatUnion |
| 146 | XZbys_LongZiSuiSui11 | China | Tibet | C | WheatUnion |
| 147 | XZbys_LongZiSuiSui17 | China | Tibet | C | WheatUnion |
| 148 | XZbys_LongZiSuiSui23 | China | Tibet | C | WheatUnion |
| 149 | XZbys_ChaYaZheDa14 | China | Tibet | C | WheatUnion |
| 150 | XZbys_ChaYaZheDa18 | China | Tibet | C | WheatUnion |
| 151 | XZbys_ChaYaZheDa19 | China | Tibet | C | WheatUnion |
| 152 | XZbys_ChaYaZheDa32 | China | Tibet | C | WheatUnion |
| 153 | XZbys_JiaChaZheDa55 | China | Tibet | C | WheatUnion |
| 154 | XZbys_JiaChaZheDa63 | China | Tibet | C | WheatUnion |
| 155 | XZbys_LangXianZheDa3 | China | Tibet | C | WheatUnion |
| 156 | XZbys_LangXianZheDa4 | China | Tibet | C | WheatUnion |
| 157 | XZbys_LangXianZheDa20 | China | Tibet | C | WheatUnion |
| 158 | XZbys_LangXianZheDa35 | China | Tibet | C | WheatUnion |
| 159 | XZbys_LongZiZheDa4 | China | Tibet | C | WheatUnion |
| 160 | XZbys_LongZiZheDa9 | China | Tibet | C | WheatUnion |
| 161 | XZbys_LongZiZheDa19 | China | Tibet | C | WheatUnion |
| 162 | XZbys_SangRiZheDa21 | China | Tibet | C | WheatUnion |
| 163 | XZD_RiKaZeLaSa1301 | China | Tibet | C | WheatUnion |
| 164 | XZD_QuShui1316 | China | Tibet | C | WheatUnion |
| 165 | XZD_GongGa1348 | China | Tibet | C | WheatUnion |
| 166 | XZD_JiangZi1405 | China | Tibet | C | WheatUnion |
| 167 | XZD_NaiDongQJ1494 | China | Tibet | C | WheatUnion |
| 168 | XZD_ZeDang1511 | China | Tibet | C | WheatUnion |
| 169 | XZD_GongBuJiangDa1523 | China | Tibet | C | WheatUnion |
| 170 | XZD_ZeDang1516 | China | Tibet | C | WheatUnion |
| 171 | XZD_ZeDang1513 | China | Tibet | C | WheatUnion |
| 172 | XZD_RiKaZeLaSa1373 | China | Tibet | C | WheatUnion |
| 173 | XZD_RiKaZeLaSa1367 | China | Tibet | C | WheatUnion |
| 174 | XZD_RiKaZeLaSa1297 | China | Tibet | C | WheatUnion |
| 175 | XZD_QuShui1409 | China | Tibet | C | WheatUnion |
| 176 | XZD_QuShui1340 | China | Tibet | C | WheatUnion |
| 177 | XZD_QiongJie1483 | China | Tibet | C | WheatUnion |
| 178 | XZD_NaiDong1503 | China | Tibet | C | WheatUnion |
| 179 | XZD_NaiDong1500 | China | Tibet | C | WheatUnion |
| 180 | XZD_MoZhuGK2065 | China | Tibet | T | WheatUnion |
| 181 | XZD_MoZhuGK2064 | China | Tibet | T | WheatUnion |
| 182 | XZD_MoZhuGK2050 | China | Tibet | T | WheatUnion |
| 183 | XZD_LaSa1342 | China | Tibet | C | WheatUnion |
| 184 | XZD_JiangZi1407 | China | Tibet | C | WheatUnion |
| 185 | XZD_GongGa1508 | China | Tibet | C | WheatUnion |
| 186 | XZD_GongBuJiangDa1526 | China | Tibet | C | WheatUnion |
| 187 | XZD_GongBuJiangDa1525 | China | Tibet | C | WheatUnion |
| 188 | XZD_DuiLongDeQing1521 | China | Tibet | C | WheatUnion |
| 189 | XZD_DuiLongDeQing1284 | China | Tibet | C | WheatUnion |
| 190 | XZbys_LangXianSuiSui6 | China | Tibet | C | WheatUnion |
| 191 | XZbys_LangXianSuiSui18 | China | Tibet | C | WheatUnion |
| 192 | XZbys_ChaYaZheDa49 | China | Tibet | C | WheatUnion |
| 193 | XZbys_JiaChaZheDa28 | China | Tibet | C | WheatUnion |
| 194 | XZbys_JiaChaZheDa37 | China | Tibet | C | WheatUnion |
| 195 | XZbys_JiaChaZheDa39 | China | Tibet | C | WheatUnion |
| 196 | XZbys_LangXianZheDa5 | China | Tibet | C | WheatUnion |
| 197 | XZbys_LangXianZheDa6 | China | Tibet | C | WheatUnion |
| 198 | XZbys_LangXianZheDa16 | China | Tibet | C | WheatUnion |
| 199 | XZbys_LangXianZheDa17 | China | Tibet | C | WheatUnion |
| 200 | XZbys_LangXianZheDa25 | China | Tibet | C | WheatUnion |
| 201 | XZbys_LongZiZheDa15 | China | Tibet | C | WheatUnion |
| 202 | XZbys_LongZiZheDa22 | China | Tibet | C | WheatUnion |
| 203 | XZbys_LongZiZheDa23 | China | Tibet | C | WheatUnion |
| 204 | XZbys_LongZiZheDa26 | China | Tibet | C | WheatUnion |
| 205 | XZbys_LongZiZheDa28 | China | Tibet | C | WheatUnion |
| 206 | XZbys_LongZiZheDa32 | China | Tibet | C | WheatUnion |
| 207 | XZbys_LongZiZheDa34 | China | Tibet | C | WheatUnion |
| 208 | XZbys_LongZiZheDa48 | China | Tibet | C | WheatUnion |
| 209 | XZbys_LongZiZheDa57 | China | Tibet | C | WheatUnion |
| 210 | XZbys_LongZiZheDa62 | China | Tibet | C | WheatUnion |
| 211 | XZbys_SangRiZheDa3 | China | Tibet | C | WheatUnion |
| 212 | XZbys_SangRiZheDa4 | China | Tibet | C | WheatUnion |
| 213 | XZbys_SangRiZheDa7 | China | Tibet | C | WheatUnion |
| 214 | YC_XZZangDong4 | China | Tibet | T | WheatUnion |
| 215 | YC_XZRiKaZe54 | China | Tibet | C | WheatUnion |
| 216 | YC_XZRiKaZe8 | China | Tibet | C | WheatUnion |
| 217 | XZD_ZhaHong | China | Tibet | C | WheatUnion |
| 218 | XZD_MoTuo | China | Tibet | C | WheatUnion |
| 219 | XZD_BianBaChunMai6 | China | Tibet | C | WheatUnion |
| 220 | XZD_WuJiangZhuo | China | Tibet | C | WheatUnion |
| 221 | XZD_MoZongZhuoGa | China | Tibet | T | WheatUnion |
| 222 | XZbys_ChaYuSuiSui2 | China | Tibet | C | WheatUnion |
| 223 | XZbys_ChaYuSuiSui3 | China | Tibet | C | WheatUnion |
| 224 | XZbys_ChaYuSuiSui5 | China | Tibet | C | WheatUnion |
| 225 | XZbys_ChaYuZheDa5 | China | Tibet | C | WheatUnion |
| 226 | XZbys_ChaYuZheDa6 | China | Tibet | C | WheatUnion |
| 227 | XZbys_ChaYuZheDa8 | China | Tibet | C | WheatUnion |
| 228 | XZbys_JiaChaZheDa61 | China | Tibet | C | WheatUnion |
| 229 | XZbys_LangXianZheDa38 | China | Tibet | C | WheatUnion |
| 230 | XZbys_LangXianZheDa47 | China | Tibet | C | WheatUnion |
| 231 | XZbys_LongZiZheDa41 | China | Tibet | C | WheatUnion |
| 232 | XZbys_LongZiZheDa42 | China | Tibet | C | WheatUnion |
| 233 | DF_XJM1 | China | Xinjiang | C | WheatUnion |
| 234 | DF_XJM20 | China | Xinjiang | C | WheatUnion |
| 235 | DF_XJM29 | China | Xinjiang | C | WheatUnion |
| 236 | DF_XJM81 | China | Xinjiang | C | WheatUnion |
| 237 | DF_XJM90 | China | Xinjiang | C | WheatUnion |
| 238 | DF_XJHongChunMai | China | Xinjiang | T | WheatUnion |
| 239 | DF_XJHongDongMai | China | Xinjiang | C | WheatUnion |
| 240 | DF_XJHongJinBaoYin | China | Xinjiang | C | WheatUnion |
| 241 | DF_YNTieKe2 | China | Yunnan | C | WheatUnion |
| 242 | DF_YNTieKe31 | China | Yunnan | T | WheatUnion |
| 243 | DF_YNTieKe32 | China | Yunnan | C | WheatUnion |
| 244 | DF_YN098 | China | Yunnan | T | WheatUnion |
| 245 | DF_YN109 | China | Yunnan | C | WheatUnion |
| 246 | DF_YN156 | China | Yunnan | C | WheatUnion |
| 247 | DF_YN411 | China | Yunnan | T | WheatUnion |
| 248 | YC_ShiLuan02d1 | China | Hebei | C | WheatUnion |
| 249 | LR_China1 | China | Hebei | C | WheatUnion |
| 250 | YC_Shi4185 | China | Hebei | C | WheatUnion |
| 251 | Shijiazhuang8 | China | Hebei | T | Laboratory |
| 252 | YCL_ShiJiaZhuang54 | China | Hebei | C | WheatUnion |

**Table S5.** The information of the wheat diversity panel and their genotypes of *TaSPP-5A* gene in the different decades

| Number | Accession | Years | Allele | Source |
| --- | --- | --- | --- | --- |
| 1 | DF_GSHongQiMai | 1904 | T | WheatUnion |
| 2 | Quality (BiYuMai) | 1924 | T | WheatUnion |
| 3 | ZhongGuoChun | 1932 | C | WheatUnion |
| 4 | Mentana (NanDai2419) | 1939 | T | WheatUnion |
| 5 | Early Premium (ZaoYangMai) | 1945 | C | WheatUnion |
| 6 | BiMa1Hao | 1947 | T | WheatUnion |
| 7 | BiMa4Hao | 1947 | C | WheatUnion |
| 8 | XiNong6028 | 1947 | T | WheatUnion |
| 9 | Abbondanza (ABo) | 1950 | C | WheatUnion |
| 10 | YouZiMai | 1950 | C | WheatUnion |
| 11 | MaZhaMai | 1950 | C | WheatUnion |
| 12 | DF_BJYanDa1817 | 1950 | C | WheatUnion |
| 13 | YCL_NanDa2419 | 1951 | T | WheatUnion |
| 14 | Xiaofoshou | 1951 | C | WheatUnion |
| 15 | Baimaza | 1952 | C | WheatUnion |
| 16 | Funo (AFu) | 1956 | T | WheatUnion |
| 17 | NeiXiang5Hao | 1958 | C | WheatUnion |
| 18 | Orofen (OuRou) | 1959 | C | WheatUnion |
| 19 | San Pastore (ASang) | 1960 | T | WheatUnion |
| 20 | JiNan2Hao | 1960 | T | WheatUnion |
| 21 | Beijing8 | 1962 | C | WheatUnion |
| 22 | Nongda311 | 1963 | C | Laboratory |
| 23 | GanMai8Hao | 1964 | C | WheatUnion |
| 24 | YCL_ShiJiaZhuang54 | 1964 | C | WheatUnion |
| 25 | Hanxuan10 | 1965 | C | Laboratory |
| 26 | St 1472/506 (ZhengYin1Hao) | 1965 | C | WheatUnion |
| 27 | Changle5 | 1969 | C | Laboratory |
| 28 | JiNing3Hao | 1969 | T | WheatUnion |
| 29 | YCL_TaiShan1 | 1969 | C | WheatUnion |
| 30 | Aimengniu | 1970 | C | WheatUnion |
| 31 | BoAi7023 | 1970 | T | WheatUnion |
| 32 | Huining10 | 1971 | C | WheatUnion |
| 33 | JiNan9Hao | 1971 | C | WheatUnion |
| 34 | FengChan3Hao | 1971 | C | WheatUnion |
| 35 | YCL_Fan6 | 1971 | C | WheatUnion |
| 36 | Keyi29 | 1980 | C | Laboratory |
| 37 | Xifeng16 | 1980 | T | Laboratory |
| 38 | YanShi4Hao | 1981 | T | WheatUnion |
| 39 | NingChun4Hao (YongLiang4Hao) | 1981 | C | WheatUnion |
| 40 | YuMai2Hao | 1983 | T | WheatUnion |
| 41 | EEn1Hao | 1985 | T | WheatUnion |
| 42 | YC_YuMai18 | 1985 | T | WheatUnion |
| 43 | YC_Jing411 | 1987 | C | WheatUnion |
| 44 | XinKeHan9Hao | 1988 | C | WheatUnion |
| 45 | YuMai13 | 1989 | C | WheatUnion |
| 46 | ChuanMai22 | 1989 | C | WheatUnion |
| 47 | Yunhan2028 | 1990 | C | Laboratory |
| 48 | Lude1 | 1990 | T | Laboratory |
| 49 | Fengkang2 | 1990 | C | WheatUnion |
| 50 | Kefeng3 | 1990 | C | WheatUnion |
| 51 | Kashibaipi | 1990 | C | WheatUnion |
| 52 | TaiShan1Hao | 1990 | C | WheatUnion |
| 53 | AiFeng3Hao | 1990 | C | WheatUnion |
| 54 | BoNong3217 | 1990 | C | WheatUnion |
| 55 | XiaoYan6Hao | 1990 | C | WheatUnion |
| 56 | ShanNong7859 | 1990 | C | WheatUnion |
| 57 | MianYang11 | 1990 | T | WheatUnion |
| 58 | LuMai1Hao (AiMengNiu) | 1990 | C | WheatUnion |
| 59 | JiNan13 | 1990 | T | WheatUnion |
| 60 | YangMai5Hao | 1990 | T | WheatUnion |
| 61 | XiAn8Hao | 1990 | C | WheatUnion |
| 62 | LuMai14 | 1990 | C | WheatUnion |
| 63 | YuMai18 | 1990 | T | WheatUnion |
| 64 | YCL_BaiNong3217 | 1990 | C | WheatUnion |
| 65 | Jingdong8 | 1991 | C | Laboratory |
| 66 | Qingshan843 | 1991 | T | Laboratory |
| 67 | ShanNongFu63 | 1991 | T | WheatUnion |
| 68 | MianYang15 | 1991 | C | WheatUnion |
| 69 | JiMai26 | 1991 | C | WheatUnion |
| 70 | Xiaoyan6 | 1991 | C | WheatUnion |
| 71 | Yimai32 | 1992 | C | Laboratory |
| 72 | Longjian196 | 1992 | C | Laboratory |
| 73 | Xifeng20 | 1994 | T | Laboratory |
| 74 | YuMai21Hao | 1994 | T | WheatUnion |
| 75 | Pingyang27 | 1994 | C | WheatUnion |
| 76 | Beijing8686 | 1995 | C | Laboratory |
| 77 | HanDan6172 | 1995 | C | WheatUnion |
| 78 | VA_China5 | 1996 | C | WheatUnion |
| 79 | LuMai21 | 1996 | C | WheatUnion |
| 80 | JiMai30 | 1996 | T | WheatUnion |
| 81 | YC_LuMai21 | 1996 | C | WheatUnion |
| 82 | YangMai158 | 1997 | T | WheatUnion |
| 83 | FengYou3Hao (YuMai47) | 1997 | C | WheatUnion |
| 84 | VA_China7 | 1998 | C | WheatUnion |
| 85 | Changmai6001 | 1998 | T | Laboratory |
| 86 | Han4589 | 1998 | C | Laboratory |
| 87 | LuMai15 | 1998 | C | WheatUnion |
| 88 | MianYang26Hao | 1998 | C | WheatUnion |
| 89 | JinMai47 | 1998 | T | WheatUnion |
| 90 | YuMai41 | 1998 | T | WheatUnion |
| 91 | JiMai38 | 1998 | C | WheatUnion |
| 92 | GaoYou503 | 1998 | C | WheatUnion |
| 93 | JiNan16 | 1998 | C | WheatUnion |
| 94 | Dan4185 | 1999 | C | WheatUnion |
| 95 | JiNan17 | 1999 | C | WheatUnion |
| 96 | YC_Shi4185 | 1999 | C | WheatUnion |
| 97 | WenMai6Hao (YuMai49) | 2000 | T | WheatUnion |
| 98 | GaoCheng8901 | 2000 | C | WheatUnion |
| 99 | Jinmai72 | 2001 | T | Laboratory |
| 100 | YanNong19 | 2001 | C | WheatUnion |
| 101 | JiMai19 | 2001 | C | WheatUnion |
| 102 | ZhongYou9507 | 2001 | C | WheatUnion |
| 103 | Jinnong 207 | 2002 | C | Laboratory |
| 104 | YanZhan4110 | 2002 | C | WheatUnion |
| 105 | VA_China1 | 2003 | C | WheatUnion |
| 106 | Chang6878 | 2003 | C | Laboratory |
| 107 | Jinmai68 | 2003 | T | Laboratory |
| 108 | Chang6154 | 2003 | C | Laboratory |
| 109 | XiaoYan22 | 2003 | C | WheatUnion |
| 110 | ZhengMai9023 | 2003 | C | WheatUnion |
| 111 | HuaiMai20 | 2003 | C | WheatUnion |
| 112 | ZhouMai16 | 2003 | C | WheatUnion |
| 113 | JiMai20 | 2003 | C | WheatUnion |
| 114 | ChuanMai42 | 2003 | C | WheatUnion |
| 115 | LunXuan987 | 2003 | C | WheatUnion |
| 116 | BoNongAK58 | 2003 | T | WheatUnion |
| 117 | YC_LX987 | 2003 | C | WheatUnion |
| 118 | VA_China2 | 2004 | C | WheatUnion |
| 119 | Chang4640 | 2004 | C | Laboratory |
| 120 | Jimai21 | 2004 | C | Laboratory |
| 121 | Longjian294 | 2004 | C | Laboratory |
| 122 | YanNong21Hao | 2004 | C | WheatUnion |
| 123 | XuMai856 | 2004 | C | WheatUnion |
| 124 | VA_China3 | 2005 | C | WheatUnion |
| 125 | VA_China4 | 2005 | C | WheatUnion |
| 126 | Chang6452 | 2005 | C | Laboratory |
| 127 | Chang6359 | 2005 | C | Laboratory |
| 128 | Heng7228 | 2005 | T | Laboratory |
| 129 | ZhouMai18 | 2005 | C | WheatUnion |
| 130 | ZhengMai366 | 2005 | T | WheatUnion |
| 131 | XiNong979 | 2005 | C | WheatUnion |
| 132 | Chang4738 | 2006 | C | Laboratory |
| 133 | HengGuan35 | 2006 | C | WheatUnion |
| 134 | LiangXing99 | 2006 | C | WheatUnion |
| 135 | YC_JiMai22 | 2006 | C | WheatUnion |
| 136 | DanJiaZhuang8Hao | 2007 | T | WheatUnion |
| 137 | ShiLuan02-1 | 2007 | C | WheatUnion |
| 138 | ZhouMai22Hao | 2007 | C | WheatUnion |
| 139 | LiangXing66 | 2008 | C | WheatUnion |
| 140 | JiMai22 | 2010 | C | WheatUnion |
| 141 | XinMai26 | 2010 | C | WheatUnion |
| 142 | Chang8744 | 2011 | C | Laboratory |
| 143 | ZhongMai175 | 2011 | C | WheatUnion |
| 144 | Villa Glori (ZhongNong28) | 2012 | C | WheatUnion |
| 145 | FengDeCunMai5Hao | 2014 | T | WheatUnion |
| 146 | ZhongMai66 | 2014 | C | WheatUnion |
| 147 | BoNong201 | 2014 | C | WheatUnion |
| 148 | Chang6794 | 2015 | C | Laboratory |
| 149 | XuMai35 | 2015 | C | WheatUnion |
| 150 | BoNong4199 | 2017 | C | WheatUnion |
| 151 | LR_China3 | 2019 | C | WheatUnion |
| 152 | CI 12203 (GanSu96) | 1944 | C | WheatUnion |
| 153 | KeChun14 | 1972 | T | WheatUnion |
